# Omega-3 Fatty Acid Supplementation and Its Impact on Systemic Inflammation and Body Weight in Patients With Cancer Cachexia—A Systematic Review and Meta-Analysis

**DOI:** 10.3389/fnut.2021.797513

**Published:** 2022-01-31

**Authors:** Gabriela Salim de Castro, Márcia Fábia Andrade, Flaydson Clayton Silva Pinto, Jaline Zandonato Faiad, Marília Seelaender

**Affiliations:** ^1^Departamento de Biologia Celular e do Desenvolvimento, Instituto de Ciências Biomédicas, Universidade de São Paulo, São Paulo, Brazil; ^2^Departamento de Cirurgia, Cancer Metabolism Research Group, LIM 26-HC, Faculdade de Medicina da Universidade de São Paulo, São Paulo, Brazil

**Keywords:** cancer cachexia, inflammation, supplementation, omega-3 fatty acids, fish oil, EPA

## Abstract

Body weight loss and inflammation are major alterations related to cancer cachexia, an important wasting syndrome highly prevalent in many types of cancer. Nutritional components modulate inflammation in several chronic diseases. Omega-3 fatty acids (n-3) are well known for their anti-inflammatory properties. However, the effects of n-3 on cancer cachexia are still controversial. This systematic review and meta-analysis aims to evaluate the reported effects of n-3 supplementation on body weight and inflammatory markers in patients with cancer cachexia. Articles indexed in the major scientific platforms were retrieved in agreement with the Preferring Reporting Items for Systematic Reviews and Meta-Analysis (PRISMA) and 167 references were initially found. After removing duplicates and applying the inclusion and exclusion criteria, this systematic review included six studies. Using a random-effects model with 95% CI, three effect sizes were expressed as standard mean difference (SMD). No differences were found regarding the effect of n-3 on interleukin-6, C-reactive protein, and albumin levels. Body weight analysis included only two studies, devoid of robust conclusions. The low number of studies, low sample size, and great intra-variability precluded a stronger analysis. More studies evaluating n-3 supplementation in cancer cachexia are still needed.

## Introduction

Body weight loss is a frequent consequence of cancer progression, and has a great impact on the tolerance to cancer treatment, quality of life, and survival (1–3). Cachexia is a wasting syndrome characterized by systemic inflammation and weight loss and described by Fearon et al. (4) as 5% or more of body weight loss over the past 6 months in the absence of simple starvation, 2% or more of body weight loss and body mass index (BMI) lower than 20 kg/m^2^, or sarcopenia and body weight loss higher than 2% (4). This scenario is often associated with reduced food intake and increased inflammation. Many mechanisms are involved in the development of cachexia and once it is established, it cannot be fully reverted by nutritional support (4).

The underlying inflammation in cachexia is recognized not only by higher levels of C-reactive protein (CRP) or low albumin (4), but a plethora of inflammatory proteins appears also to be altered in this syndrome. Therefore, it is important to screen for other inflammatory parameters, such as cytokines, as interleukins (ILs) (5, 6). The search for a nutrient that modulates inflammation and weight loss has been a major challenge in the treatment of cachexia (7). Omega-3 polyunsaturated fatty acids (n-3) can be incorporated in the cell membrane, influencing fluidity and in long term, immunomodulation, decreasing the production of inflammatory eicosanoids, cytokines as IL-6, IL-8, and the transcription factor nuclear factor-kappa B (NFκB), an important regulator of immune response (8). Moreover, n-3 supplementation was shown to be effective in decreasing IL-6 and increasing albumin in a meta-analysis that included studies with patients with colorectal cancer (9).

Reducing the inflammatory status in cachectic patients may be the key to inducing weight stabilization and better outcome (10). Moreover, international nutritional guidelines report that n-3 supplementation seems to be safe (11). We conducted a systematic review and meta-analysis aiming to verify if n-3 supplementation contributes to reduce systemic inflammation and body weight loss in patients with cancer and cachexia.

## Methods

The systematic review and a meta-analysis were performed according to the guidelines of the Cochrane Handbook for Systematic Reviews of Interventions (12) and reported according to the Preferred Reporting Items for Systematic Reviews and Meta-Analysis (PRISMA) guidelines (13).

### Literature Search Strategy

A literature search of randomized controlled trials (RCTs) to investigate the effects of polyunsaturated fatty acid supplementation on inflammatory profile and body composition in cancer cachexia in the adult patients was performed by means of a search in 3 literature databases. Initially, with the help of the search string, a researcher (JF) performed a search in the database (last search date on August 2021) of the Web of Science, PubMed, and LILACS. No restrictions were applied to the initial electronic search. For retrieval of studies, the following descriptors were used, searched under the DeCS terms: “fish oil” or “omega 3 fatty acid” or “n-3” or “ω-3” or “ω-3 fatty acid” or “polyunsaturated fatty acid” or “PUFA n-3” or “eicosapentanoic acid” or “EPA” or “docosahexaenoic acid” or “DHA” and “supplementation” or “supplement” and “inflammation” or “interleukin-6” or “IL-6” or “C-reactive protein” or “CRP”and “muscle mass” or “skeletal muscle” or “body composition” and “cancer” and “cachexia” or “sarcopenia” or “weight loss.”

### Eligibility Criteria

After removing duplicates and irrelevant material, the titles and abstracts identified in the search were independently selected by two investigators (FP and JF). Potentially eligible studies had their full texts selected by the same two investigators (FP and JF). Disagreements among reviewers were discussed and decided by the consensus of all the authors.

The selected studies followed the inclusion criteria based on the patient, intervention, comparison, and outcome (PICOS) strategy. As shown in Table 1, the inclusion criteria comprised: polyunsaturated fatty acid supplementation was offered and described; measurement of inflammatory markers, of body weight, and body composition in cancer cachectic patients (any tumor), no restriction of the year of publication, works published and available in full within the science platforms sought. The exclusion criteria encompassed: studies that did not use n-3, that were not performed with patients with cancer cachexia or with previous weight loss; studies using experimental and/or *in vitro* models; non-adult individuals; literature review and studies that did not present inflammatory data.

**Table 1:** Inclusion and exclusion criteria performed by patient, intervention, comparison, and outcome (PICOS) strategy.

	**Inclusion criteria**	**Exclusion criteria**
Population	Adult (>18 y) with a clinical diagnosis of cancer cachexia	Adult (<18 y); non-cancer cachectic patients
Intervention	Any isolated omega-3 supplementation or in combination with other nutrients of any duration	No omega-3 supplementation
Counterpart	Any counterpart group	None
Outcomes	Inflammatory profile and body composition (measured by any means)	None
Study design	Randomized controlled trial	Nonrandomized controlled trial

### Extraction and Synthesis of Data

Data extraction was independently performed by 4 investigators (FP, GC, JF, and MA) using a pre-specified data collection form, cross-checked for discrepancies, and corrected when appropriate (FP and MA). The data extraction from each study was performed as follows: (a) general information about the selected study (i.e., authors, journal, and year of publication); (b) information about the type of intervention and respective control counterparts; (c) population included and information about parameters analyzed in the study, (d) how studies dealt with results, (e) primary and secondary results related to the purpose of the systematic review, (f) discussion, and (g) data sources used in the study.

### Assessing the Quality of Trials

Four investigators (FP, GC, JF, and MA) assessed the quality of evidence and the risk of bias in each study using “the Cochrane Collaboration's” tool (14). In this study, the application of this quality tool was independently organized and differences were resolved by consensus. The tool establishes three levels of classification for the eight items: “Low bias” reported complete information, “Unclear bias” partially or with indistinct reported information, and “high bias” for unreported information (14). Trials were considered low risk when information on allocation concealment, blinding of participants and researchers, compliance assessment performed, number of dropouts, and reported reasons were presented. Otherwise, trials were considered to be at high risk of bias or with unclear bias if the risk of bias could not be determined or unidentified, respectively.

### Statistical Analysis of Data

Meta-analyses were performed on the extracted data, where applicable, using a random-effects model in the Review Manager version 5.4.1 (RevMan) (15). Initially, data were organized and standardized by one investigator (FP), to facilitate the analysis. Data from studies that had multiple time points had the last one included and compared to the baseline in the overall meta-analysis. For the statistical analysis, data extracted from the selected studies were standardized to obtain mean and SD.

Due to the difference in data reporting (values at the end of the intervention or differences after the intervention), the variation from baseline was calculated from the available data according to the Cochrane Handbook for Systematic Reviews of Intervention (12). When reported, the data presented in mean and standard error (SE), median and interquartile range (IQR), median and Range and point estimate with 95% CI were calculated using specific formulas (16) and from the available data, and SDs imputed according to the Cochrane Handbook for Systematic Reviews of Interventions (12). Subgroup analysis was performed to explore the effect of n-3 supplementation depending on the route of administration (i.e., oral route or feeding tube) on the overall outcome. The standard mean difference (SMD) with 95% CIs were used to express the effect size estimates, with SMD values of ≤ 0.2, considered as having a low effect, 0.3–0.5 as a moderate effect, and >0.5 were defined as large effect size, due to the different methods used to assess inflammatory markers and body weight data (12).

Finally, the heterogeneity of results between studies was determined by *I*^2^, where ≤ 49.9% were considered low values, 50–74.9% medium, and 75–100% as high heterogeneity. The z-score was used as a general effect test, considering *p* ≤ 0.05 as significant. As recommended by the Cochrane Handbook for Systematic Reviews of Interventions (12) and its clinical relevance, effect sizes were also considered.

## Results

### Study Identification and Selection

**Figure 1:**
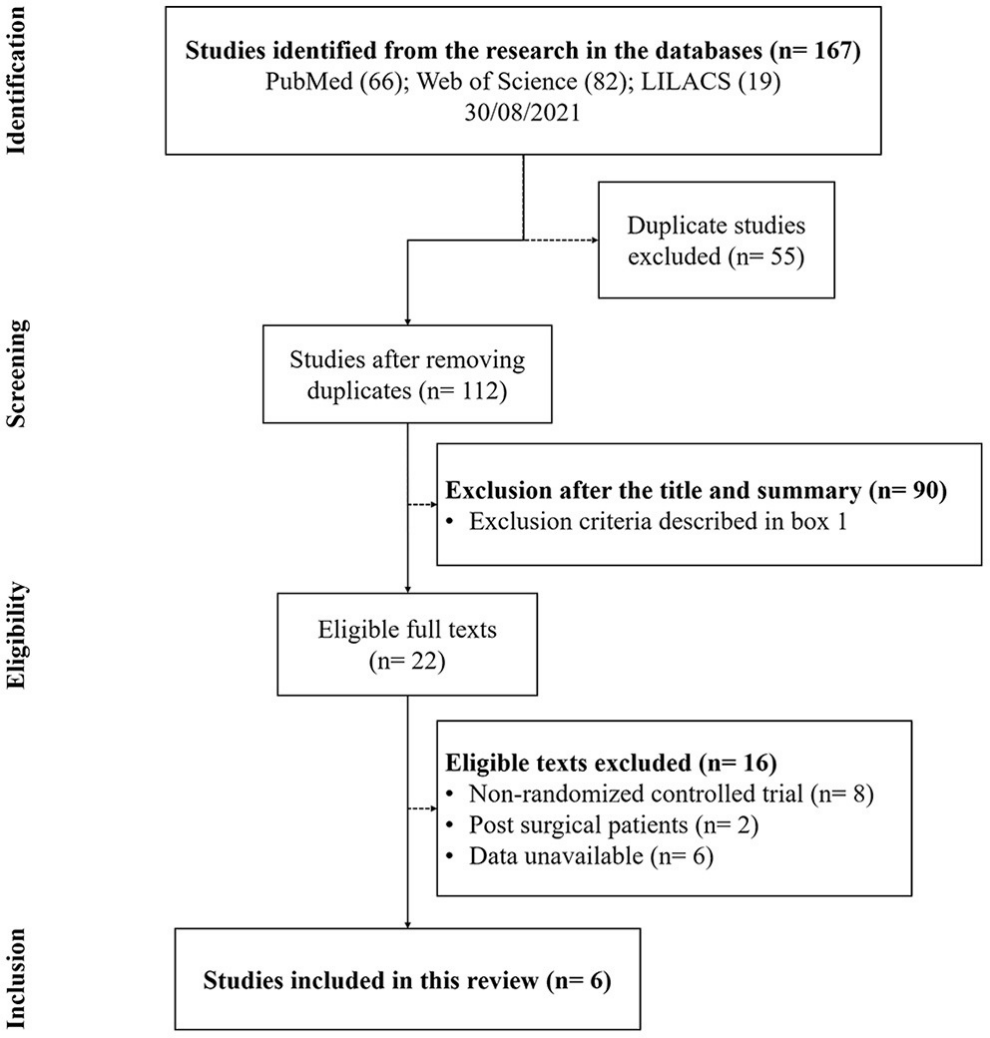
PRISMA flowchart of studies evaluated and included.

In total, 167 references were found. After removing the duplicates (55), 112 articles remained. Of these 35 review articles, two proceeding papers, eight in languages other than English and 61 were excluded after reading the title and abstracts because they failed to match the inclusion criteria (Box 1). Six studies remained, which were included in this systematic review (Figure 1) (13). The main characteristics are shown in Table 2.

Box 1 **Excluded reasons of studies.**

**Table:** 

**Exclusion criteria**	***n***
1. No n-3 polyunsaturated fatty acid supplementation	3
2. Non-cancer cachectic patients	14
3. Reviews	35
4. Pharmacological associated treatment	6
5. Total parenteral nutrition or intravenous nutrient infusion	2
6. Animal studies	19
7. *In vitro* studies	1
8. Other languages studies	8
9. Other types of articles (proceeding paper)	2

**Table 2:** Main characteristics of the studies included in the meta-analysis.

**Study**	**Subjects^a^**	**Age (years)^b^**	**Type of study**	**Intervention**	**Study design**	**Duration of intervention**	**Main outcomes**	**Other outcomes**
**Oral supplementation**								
Carvalho et al. (17)	(I) 29 (4) (C) 24 (7) Oral cavity cancer	(I) 53.3 ± 8.8 (C) 57.3 ± 9.1	Randomized, controlled clinical trial	Hypercaloric and hyperproteic supplement with 2 g EPA/440 mL	135 g/day of hypercaloric and hyperproteic supplement	4 weeks	The supplementation was not able to promote significant changes in the inflammatory profile	The EPA group presented 50% less likelihood of nutritional risk according to the CRP/Albumin ratio, despite not having shown any statistical difference.
Faber et al. (18)	(I) 24 (7) (C) 23 (7) Adenocarcinoma or squamous carcinoma in the esophagus or gastroesophageal junction	(I) 61.1 ± 9.2 (C) 61.6 ± 9.4	Explorative, randomized, controlled, double-blind study	400 mL/day.652 kcal, 39.6 g ptn, 2.4 g EPA, 1.2 g DHA, 4.8 g GOS, 0.8 g FOS and a balanced mix of vitamins, minerals	400 mL per day, 0–5% WL, a non-caloric Placebo product ≥5% WL, isocaloric standard nutritional product	4 weeks	A significant increase in body weight and an improved performance status in patients who received the nutritional intervention with EPA/DHA, which is high in protein and leucine.	There was a significantly higher decrease in the ratio n-6/n-3 compared to the control group.
Liu et al. (19)	(I) 11 (C) 11 Gastric cachexia cancer	(I) 56 (49–75)^3^ (C) 58 (45–75)^3^	Randomized study	3.6 g of *n*-3/day	6 mL of atractylenolideI (ATR)/day	6 weeks	N-3 group showed lower serum values of IL-6. ATR was more effective than FOE in improving appetite and, Karnofsky performance.	ATR decreased the proteolysis- inducing factor in urine.
Persson et al. (20)	(I) 13 (6) (C) 11 (4) Advanced gastrointestinal cancer	(I) 66 ± 9 (C) 69 ± 10	One-center, randomized, non–placebo-controlled, open study	30 mL/day of FO mixture - 4.9 g of EPA and 3.2 g of DHA	18 mg of Melatonin (MLT)/day	4 weeks	FO, MLT did not demonstrate anti-inflammatory effect.	No differences were observed in serum albumin, CRP, TNF-α, IL-1β, soluble IL-2 receptor, IL-6, IL-8 and plasma fibrin.
**Enteral nutritional supplementation**								
Solís-Martínez et al. (21)	(I) 32 (14) (C) 32 (15) HNC squamous cell cancer in cancer treatment	(I) 60 ± 14 (C) 58 ± 14	A randomized single-blind placebo-controlled	A high-protein supplement with 2 g of EPA per day (600 kcal, 40 g of ptn)	A high-protein supplement with 24 g calcium caseinate per day (596 kcal, 40 g of ptn)	6 weeks	EPA supplement was associated with BW and LBM stabilization.	There was a significant increase in IL-8 levels and decreased of fatigue.
Yeh et al. (22)	(I) 31 (1) (C) 37 (0) HNC in cancer treatment	(I) 54.1 ± 9.3 (C) 54.4 ± 9.8	A randomized, prospective, clinical trial	An energy dense oral nutritional supplement with 7.1 g of n-3 and glutamine, probiotics and vitamins.	Isocaloric nutrition formulation	8 weeks	The supplementation improved BW, serum albumin and, prealbumin levels in patients with BMI <19.	Severe diarrhea events reported in the intervention group could be related to the higher osmolarity and different fat content of the formula.

a *Female gender is in brackets*.

b *Values are mean ± SD unless otherwise specified*.

*I, intervention group; C, Counterpart group; EPA, eicosapentanoic acid; DHA, docosahexaenoic acid; CRP, C-reactive protein; HNC, head and neck cancer; Ptn, protein; GOS, galacto-oligosaccharides; FOS, fructo-oligosaccharides; WL, weight loss; ATR, atractylenolide; FO, fish oil; MLT, melatonin; BW, body weight; LBM, lean body mass; BMI, body mass index*.

*^2^Completed patients*.

3 *Point estimation 95%CI*.

*^4^Median (range)*.

*^5^Median (IQR)*.

### Study Characteristics

The 6 eligible RCTs involved a total of 278 patients (17–22). Three out of the six selected studies enrolled patients with head and neck cancer (HNC) (17, 21, 22) the other 3 inscribed patients with gastrointestinal cancer (18–20). Only two studies included patients under tube feeding regimen and both also reported cancer treatment during supplementation (21, 22). The duration of the intervention was between 4 and 8 weeks and the n-3 amount offered was of between 2 and 7.1 g/day. Table 3 shows the baseline data from all the studies included.

**Table 3:** Baseline characteristics of the subjects from studies included in the meta-analysis.

**Study**	**Previous weight loss (%)**	**Body weight (kg)**	**Weight variation (kg)**	**BMI (kg/m^2^)**	**Lean mass (kg)**	**IL 6 (pg/mL)**	**CRP (mg/L)**	**Albumin (g/L)**
**Oral supplementation**								
Carvalho et al. (17)	(I) 13.0 ± 8.9 (C) 14.2 ± 5.0	(I) 55.8 (37.5–100.0)^3^ (C) 57.2 (40.3–80.1)^3^	NR	(I) 20.7 ± 3.4 (C) 22.6 ± 4.3	NR	(I) 2.58 (0–16.97)^3^ (C) 2.17 (0–14.34)^3^	(I) 0.41 (0.04–50.55)^3^ (C) 1.46 (0.06–20.11)^3^	(I) 4.40 (3.20–4.80)^3^ (C) 4.30 (2.70–5.20)^3^
Faber et al. (18)	(I) −4.2 ± 6.0 (C) −3.8 ± 5.7	NR	NR	(I) 25.5 ± 4.6 (C) 25.4 ± 3.6	NR	(I) 0.0 (−0.5–1.2)^4^ (C) 0.0 (−1.5–0.1)^3^	(I) 0.5 (0.0–3.3)^4^ (C) 0.0 (−2.3–1.5)^4^	NR
Liu et al. (19)	(I) −0.10 to 0.07^*^^2^^2^ (C) −0.12 to 0.10^*^^2^^2^	NR	NR	NR	NR	(I) 113.11 to 126.29^2^^2^ (C) 113.64 to 128.96^2^^2^	NR	NR
Persson et al. (20)	(I) −13.2 ± 8.4 (C)-10.8 ± 8	(I) 56.6 (35–101)^3^ (C) 61.8 (33–80)^3^	(I) −0.6^*^ (C) 1.8^*^	(I) 21.6 ± 4.1 (C) 21.1 ± 4.8	NR	(I) 4.7 (1.5–7.6)^3^ (C) 4.9 (1.4–18.9)^3^	(I) 22.5 (10–124) (C) 633 (10–229)	(I) 39 (27–48)^3^ (C) 34 (27–40)^3^
**Enteral nutritional supplementation**								
Solís-Martínez et al. (21)	(I) 13.33 ± 8.10 (C) 13.74 ± 9.27	(I) 58.8 ± 14.0 (C) 61.1 ±11.5	(I) −0.3 ± 5.9 (C) −2.1 ± 3.7	(I) 22.6 ± 4.6 (C) 24 ± 4.2	(I) 39.4 ± 9.6 (C) 42.6 ± 9.5	(I) 333 ± 309.8 (C) 196.8 ± 256.4	(I) 21 ± 35 (C) 25 ± 38	(I) 3.79 ± 0.67 (C) 3.66 ± 0.49
Yeh et al. (22)	NR	(I) 54.9 ± 9.4 (C) 54.5 ± 10.8	(I) 1.12 ± 8.03^#^ (C) −6.30 ± 9.41^#^	(I) 20.0 ± 3.1 (C) 19.8 ± 3.7	NR	NR	NR	(I) 3.2 ± 0.5 (C) 3.3 ± 0.5

*^1^Values are mean ± SD unless otherwise specified*.

*I, intervention group; C, Counterpart group; BMI, body mass index; NR, not reported*.

2 *Estimated point 95%CI*.

3 *Median (range)*.

4 *Median (IQR)*.

* *Values are kilograms (kg)*.

# *Values are percentage (%)*.

### Risk of Bias

**Figure 2:**
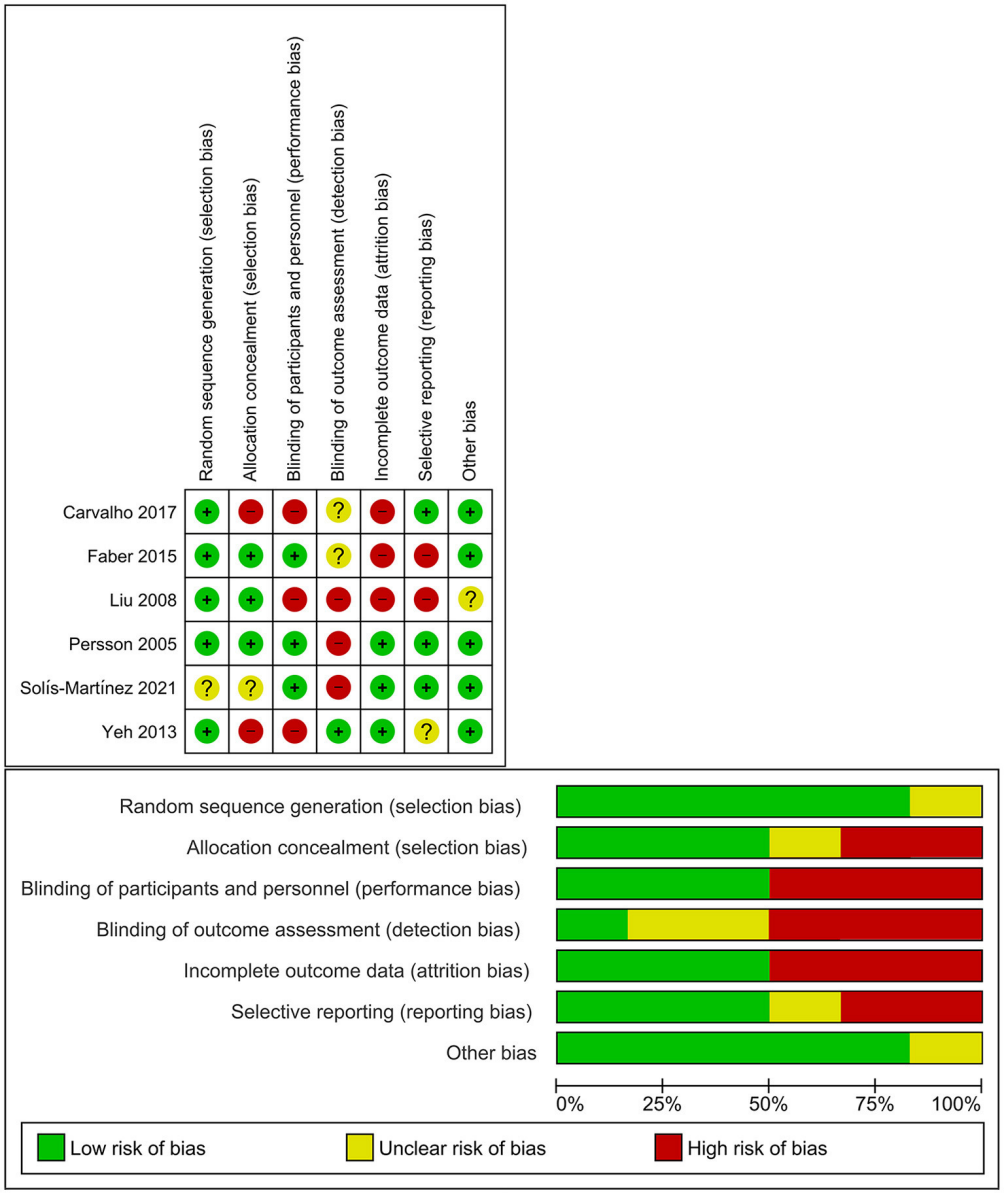
Risk of bias from review authors' judgments of each study and presented as a percentage across the included studies.

The risk of bias is compiled in Figure 2. Most of the studies had a low risk of bias for the random sequence generation (17–20, 22), allocation concealment (18–20), and blinding of participants and personnel-to-random allocation (18, 20, 21). The blinding of outcome assessment presented a high risk of bias (19–21), as only one study was double-blind (18), while one study was single-blind (21). Half of the studies showed incomplete data outcomes (17–19) indicating that not all the proposed analyses were presented in the article as results. Also, two studies had a high risk of reporting bias due to selective reporting (18, 19). Only one of them had an unclear risk of other bias (19).

### Body Composition and Inflammatory Profile

**Figure 3:**
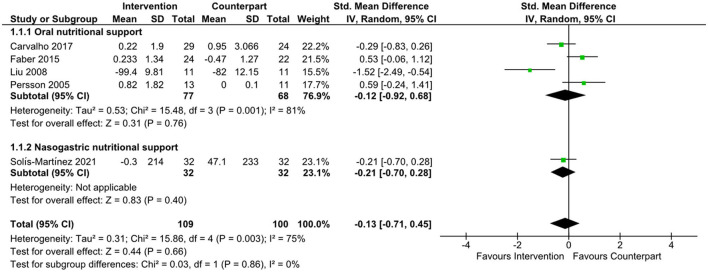
Forest plot of standard mean difference in interleukin 6 levels.

**Figure 4:**
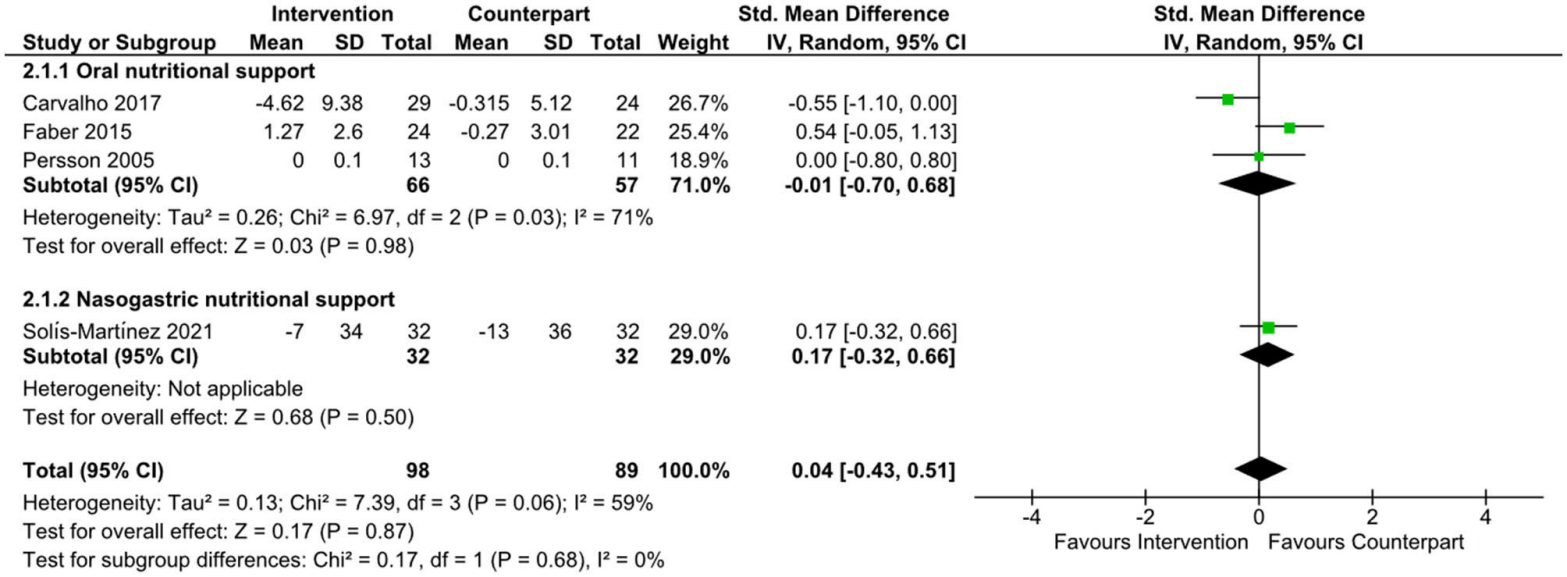
Forest plot of standard mean difference in C-reactive protein levels.

**Figure 5:**
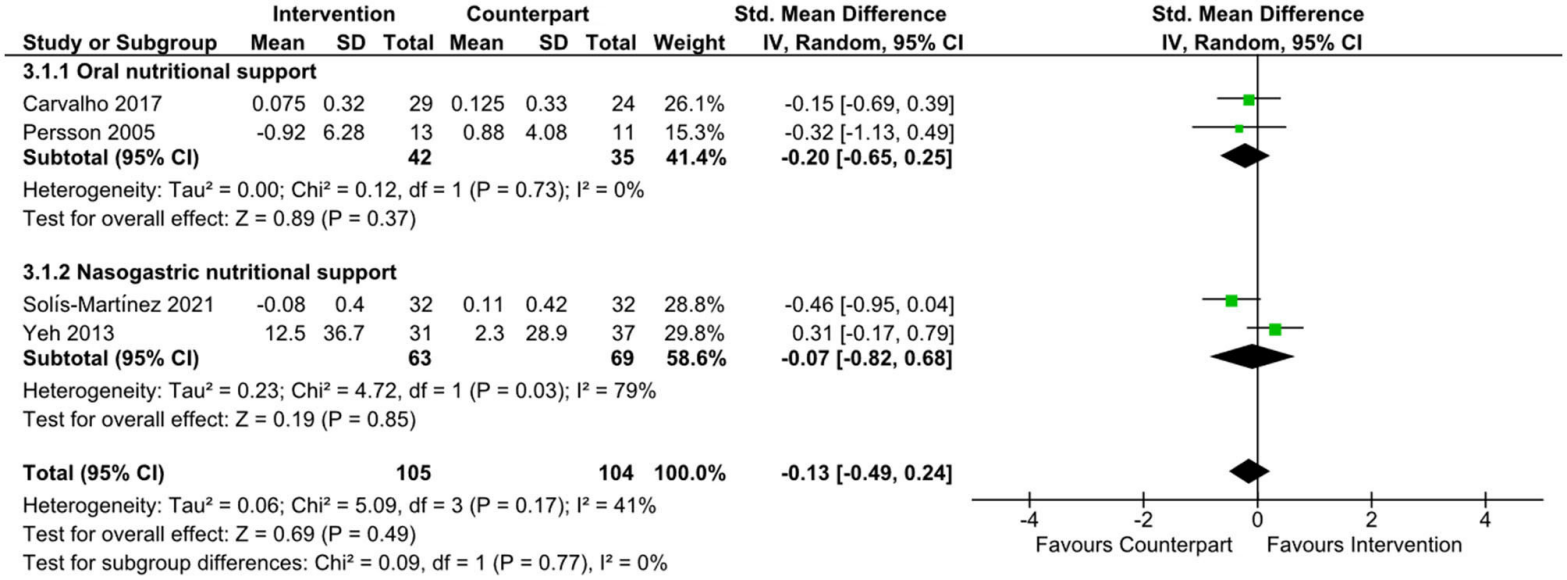
Forest plot of standard mean difference in albumin levels.

There was an improvement in body weight favoring n-3 supplementation (21, 22) (data not shown) in the overall meta-analysis (SMD = 0.60; 95% CI: 0.13, 1.07; *z* = 2.50; *p* = 0.01). Subgroup analyses were not undertaken due to the small number of studies. There was no evidence to support the effect of n-3, or supplements containing n-3 on changes in IL-6, as shown in Figure 3 (SMD = −0.13; 95% CI: −0.71, 0.45; *z* = 0.44; *p* = 0.66), CRP (Figure 4, SMD = 0.04; 95% CI: −0.43, 0.51; *z* = 0.17; *p* = 0.87), and albumin (Figure 5, SMD = −0.13; 95% CI: −0.49, 0.24; *z* = 0.69; *p* = 0.49) in the overall meta-analysis. Subgroup analyses were not performed due to the small number of studies fulfilling the criteria of the present review. The overall meta-analysis heterogeneity *I*^2^ of IL-6, CRP, and albumin analysis was 75, 59, and 41%, which is considered high, medium, and low, respectively. Table 4 presents the main findings of the studies included in the meta-analysis.

**Table 4:** Main findings of the included studies.

**Author**	**Duration (weeks)**	***n*-3 (g/day)**	**Main change in inflammation**
Carvalho et al. (17)	4	2	-
Faber et al. (18)	4	3.6	↓ PGE2
Liu et al. (19)	6	3.6	↓ IL-6
Persson et al. (20)	4	8.1	-
Solís-Martínez et al. (21)	6	2	↑ IL-8
Yeh et al. (22)	8	7.1	↑ Albumin

*↑ Increased, ↓ Decreased*.

## Discussion

Eicosapentanoic acid and DHA are long-chain omega-3 polyunsaturated fatty acids (n-3) that present anti-inflammatory and immune modulation properties and have been extensively studied in the context of cancer (22). A decrease in inflammation could potentially cause an improvement in food intake, a reduction in the rate of body weight loss, and consequently improve quality of life (23). The promising benefits of n-3 in counteracting inflammation have been demonstrated clearly in various chronic diseases. Nevertheless, in the scenario of cancer-related inflammation, we could not detect a robust reduction in IL-6 and CRP levels in the patients with cancer and cachexia after n-3 supplementation. These findings may be due to the low number of studies that matched the inclusion and exclusion criteria and/or the lack of n-3 capability in decreasing inflammation in patients with cancer cachexia.

The enzymes cyclooxygenase and lipoxygenase catalyze the synthesis of prostaglandins and other eicosanoids from omega-6 (n-6) and n-3 fatty acids located in the cellular membrane (24). These metabolites have a more prominent pro-inflammatory activity when derived from n-6 and more resolution-driven action when are originated from n-3 (25). Increased intake of n-3 results in higher incorporation of this fatty acid and lower content of n-6 in plasma membranes (8). Moreover, n-3 are able to bind to the membrane or to intracellular receptors and inhibit pro-inflammatory transcription factors, as NFκB, which regulates the synthesis and secretion of IL-6. This cytokine is the main stimulus for CRP secretion by the liver. Hampering NFκB through n-3 intake would result in lower IL-6 and pro-inflammatory eicosanoid levels (8).

Our meta-analysis did not find a decrease in IL-6 and CRP levels after n-3 supplementation in patients with cancer cachexia. It has been suggested that n-3 incorporation is dose and time-depended (26), therefore, lower doses would need more time to be effective. The studies included in this meta-analysis have a great variety of doses and duration. The longest supplementation combined with the highest dose showed an improvement in serum albumin, which could implicate a decrease in CRP; however, this study did not measure inflammatory markers (22). Supplementation of 3.6 g n-3/day during 4 weeks decreased PGE_2_, but no differences were observed for IL-6 levels (18). The supplementation of 3.6 g/day of n-3 during 6 weeks lowered IL-6 levels when compared to a traditional Chinese medicine supplementation (19). The other studies could not demonstrate any benefits of n-3 supplementation.

The study by Carvalho et al. (17) investigated the effect of nutritional supplementation employing a nutritional formula enriched with EPA on the inflammatory profile of patients with cancer in the oral cavity (tongue, floor, and gums in stages III and IV). The study randomized 53 patients into 2 groups: the intervention group, who received a hyper-caloric, high-protein supplement with 2 g EPA, and the control group, who received an isocaloric isoproteic powdered placebo, both for 4 weeks. None of the groups experienced side effects during the intervention. There were no significant differences in IL-6, albumin, pre-albumin, and CRP between those who received standard supplement and EPA-enriched supplement group. Some biases were observed, as the study was not blind and, the sample size, small. Moreover, the study reported low adherence to nutritional supplementation, as the compliance was around 80% in both groups, which may have influenced the absence of changes in inflammatory markers in the intervention group (17).

The effect of nutritional intervention in newly diagnosed esophageal cancer patients was investigated by Faber et al. (18). Patients were randomized into two groups that received a high-protein compound enriched with 2.4 g of EPA and 1.2 g of DHA per day (intervention group) or an isocaloric or noncaloric placebo product depending on the weight loss: ≥5 or up to 5 %, respectively (Counterpart group), for 4 weeks. In addition, 40 healthy volunteers, without intervention, were included for comparison. Patients of the intervention group demonstrated a significant increase in body weight and an improvement in the performance status when compared with the control group. A possible explanation regarding the higher body weight could be due to the high protein (39.6 g/day) and leucine (4.4 g/day) content in the supplement offered to the intervention group (27, 28). The trial showed a significant decrease in serum prostaglandin E_2_ (PGE_2_) levels in the intervention group compared to the counterparts. These differences were more pronounced when groups were stratified by weight loss, showing that patients in the intervention group with ≥5 % weight loss had a more pronounced decrease in PGE_2_ (18).

Furthermore, Faber et al. (18) also showed the incorporation of EPA and DHA fatty acids in plasma phospholipids comparing these same groups and there was a significant increase of these compounds in the intervention group. It is important to consider that total caloric intake was not assessed and differences in food consumption could have contributed to weight gain ascribed to the intervention group. Moreover, the n-3-rich supplement was composed of different bioactive compounds that may have influenced the results (18).

In another study, the administration of n-3 was compared to atractylenolide I (ATR), the bioactive compound with anti-inflammatory properties extracted from atractylodes rhizome, a plant used in traditional Chinese medicine to treat anorexia and other gastrointestinal tract symptoms (19). Both the supplements were offered to patients with gastric cancer and cachexia. The n-3 group was supplemented in 8 capsules containing 3.6 g of fish oil per day (2.52 g of EPA + DHA per day) and the ATR group received 12 mL of extracts from atractylodes rhizome (containing 0.11 g/mL of ATR I). Both groups received treatment for 6 weeks with a 1-week interval after the 3rd week. This study also collected blood from 11 healthy volunteers and serum was used to compare the pre and posttreatment cytokine content of patients with gastric cancer. ATR I was more effective in improving Karnofsky Performance Score and appetite compared to the n-3 group. With respect to inflammatory parameters, the n-3 group showed lower serum values of IL-6 after 6 weeks of supplementation, as compared to the ATR group. N-3 supplementation was also able to decrease the levels of IL-1 over the 6 weeks and the cytokine concentration posttreatment values were not different from the healthy control group. It is important to note that the study of Liu et al. (19) had a low number of patients per group and an unusual way to report data, as point estimate and confidence interval; however, it was still able to show that n-3 supplementation decreased inflammation in patients with gastric cancer and cachexia (19).

The highest dose of n-3 administered in the studies included in this meta-analysis was offered in the study of Persson et al. (20). The effect of 30 mL of a fish oil mixture (total of 4.9 g of EPA and 3.2 g of DHA) per day (fish oil—FO group) was compared to 18 mg per day of melatonin (melatonin group), offered for 4 weeks to patients with advanced gastrointestinal cancer, not amenable to curative or palliative treatment and with weight loss >10% or decreased serum albumin. After 4 weeks of supplementation, both the groups received 30 mL of fish oil together with 18 mg of melatonin per day for additional 4 weeks. Patients in the FO group had increased EPA and DHA serum levels and decreased linoleic acid levels, also showing a decrease in fatigue, and improved emotional functional score, and did not lose body weight and lean mass during the initial 4 weeks compared with the melatonin group. Although the consumption of melatonin along FO seemed to stabilize weight loss, the results were not reach statistical significance and no other benefits were observed. Only 62% of the FO group showed good compliance to the supplementation regimen and the study was not blind to allocation of patients (20).

Solís-Martínez et al. (21) compared a polymeric supplement enriched with 2 g of EPA (intervention group) to a standard polymeric supplement in patients with HNC for 6 weeks. Both supplements offered the same amount of protein and were very similar in calorie content. Patients received the supplement orally or by enteral feeding tube and were under cancer treatment. There was a significant increase in IL-8 and HDL and a decrease in lymphocytes, triglycerides, and LDL in the intervention group compared with the control group. Furthermore, the intervention group showed improved emotional function scores and also reduction of fatigue. The compliance to the supplement ingestion was 72% in the intervention group compared to 86% in the control group. Besides the better compliance and higher ingestion of calories and proteins, the control group had significant weight loss, which was not observed in the intervention group, as the latter presented body weight and lean mass stabilization over the study.

The study of Yeh et al. (22) enrolled 68 patients with HNC and cachexia that were under chemotherapy or radiotherapy. They were randomized to receive either a protein- and energy-dense supplement (290 kcal in 72 g) enriched with n-3 fatty acids (1.4 g in 72 g), glutamine, selenium, and coenzyme Q10 plus an enzyme product (pineapple and papaya enzymes) containing probiotics (8 billion) and vitamins (intervention group) or a commercial nutritional formula (250 kcal in 237 mL, control group). Patients were supplemented for 8 weeks and throughout this time 76.4 and 44.1% of the patients from the control and intervention group, respectively, needed a nasogastric tube feeding. They were instructed to consume 1,500 kcal of the nutritional supplement, which means 7.1 g of n-3 per day. The major part of the energy consumed by both groups came from nutritional supplements. It is important to note that the average energy consumption reported in this study for both groups was around 1,300 kcal/day, which would mean 6 g/day of n-3 for the intervention group (22).

The intervention group from the study of Yeh et al. (22) maintained the body weight during the first 4 weeks. After stratification by BMI, it was evident that patients with BMI <19 kg/m^2^ in the intervention group were able to increase body weight during the 8 weeks of the study and the improvement in serum albumin was higher than in those patients receiving the same supplement, but with BMI >19 kg/m^2^ and compared with patients receiving the control supplement also stratified by BMI (>19 kg/m^2^ or <19 kg/m^2^). Nevertheless, the energy-dense supplement offered to the intervention group caused more frequent severe diarrhea events and patients from both groups were not able to consume the 1,500 kcal/day from supplements, as in the last week both groups were consuming an average of 800 kcal/day that were provided completely by the supplement formulas. The study was able to demonstrate an improvement in body weight in the intervention group with lower BMI, indicating that patients with more severe cachexia may present a more robust beneficial effect from supplements with anti-inflammatory and antioxidants compounds. The drawbacks of this study include the lack of blinding of the researchers and clinical team to allocation of the patients and a low number of patients in the treatment group with BMI <19 kg/m^2^ (22).

Of the 6 studies included, only two were blind −1 double-blind (18) and 1 single-blind (21). The majority of the studies did not measure n-3 fatty acids incorporation, with the exception of Faber et al. (18) and of Persson et al. (20). Of note, the n-6/n-3 ratio should be considered during supplementation, as this ratio influences n-3 incorporation in the plasma membrane (29, 30). Therefore, the effects of n-3 supplementation in the clinical studies should be carried out with the concommitant analysis of n-6 consumption, as to assess the corresponding proportion of n-6/n-3 to be effective. A high dose of n-3 offered for 8 weeks was able to increase albumin and improve body weight in the patients with severe cachexia (22). On the contrary, the high amount of n-3 offered in the Persson et al. (20) study may have had its anti-inflammatory effects masked by the administration of n-3 to the control group in the last 4 weeks of the study. The only study that offered capsules of fish oil for 6 weeks also showed a decrease in inflammation, with a dose, which cannot be considered high (19).

### Strengths and Limitations

The meta-analysis did not show a significant improvement in inflammatory markers and body weight when patients with cancer and cachexia received n-3 supplementation. It is important to note that we only included studies that showed previous body weight loss, while reporting increased circulating inflammatory markers and/or decreased albumin and devoid of administration of anti-inflammatory drugs in the intervention. Although strict inclusion and exclusion criteria are necessary to decrease the heterogeneity, it was also the major limitation, as our analysis was confined to a few studies, narrowing sample size.

## Conclusion

N-3 supplementation alone or in nutritional formulas did not show a positive effect on the circulating inflammatory markers. Although a positive effect has been found in body weight favoring n-3, only two studies addressing this aspect were evaluated in this analysis. Thus, we cannot support this finding due to small sample size. The studies included have confounding factors, with varying doses and administration of multiple nutrients. Therefore, more studies are necessary to elucidate the role of n-3 fatty acids in decrease systemic inflammation related to cancer cachexia.

## Data Availability Statement

The raw data supporting the conclusions of this article will be made available by the authors, without undue reservation.

## Author Contributions

FP, GC, JF, and MA were involved in the study design, data collection, drafting the manuscript, contributed to data interpretation, and article writing. FP was involved in collation of results and analysis. MS helped in critically reviewing, data interpretation, and article writing. All authors read and approved the final manuscript.

## Funding

We acknowledge the São Paulo Research Foundation (FAPESP Grant 20/07765-6 and 12/50079-0 to MS) and Fundação Faculdade de Medicina for financial support.

## Conflict of Interest

The authors declare that the research was conducted in the absence of any commercial or financial relationships that could be construed as a potential conflict of interest. The reviewer AL declared a shared affiliation, though no other collaboration, with the authors GC, MA, FP, JF, and MS to the handling Editor.

## Publisher's Note

All claims expressed in this article are solely those of the authors and do not necessarily represent those of their affiliated organizations, or those of the publisher, the editors and the reviewers. Any product that may be evaluated in this article, or claim that may be made by its manufacturer, is not guaranteed or endorsed by the publisher.

## References

[1] Ryan AM Power DG Daly L Cushen SJ Ní Bhuachalla E Prado CM. Cancer-associated malnutrition, cachexia and sarcopenia: the skeleton in the hospital closet 40 years later. Proc Nutr Soc. (2016) 75:199–211. 10.1017/S002966511500419X 26786393

[2] Cespedes Feliciano EM Lee VS Prado CM Meyerhardt JA Alexeeff S Kroenke CH . Muscle mass at the time of diagnosis of nonmetastatic colon cancer and early discontinuation of chemotherapy, delays, and dose reductions on adjuvant FOLFOX: The C-SCANS study. Cancer. (2017) 123:4868–77. 10.1002/cncr.30950 28881381 PMC5716836

[3] Meyerhardt JA Kroenke CH Prado CM Kwan ML Castillo A Weltzien E . Association of weight change after colorectal cancer diagnosis and outcomes in the kaiser permanente northern california population. Cancer Epidemiol Biomarkers Prev. (2017) 26:30–7. 10.1158/1055-9965.EPI-16-0145 27986654 PMC5225080

[4] Fearon K Strasser F Anker SD Bosaeus I Bruera E Fainsinger RL . Definition and classification of cancer cachexia: an international consensus. Lancet Oncol. (2011) 12:489–95. 10.1016/S1470-2045(10)70218-7 21296615

[5] Fearon KCH Glass DJ Guttridge DC. Cancer cachexia: mediators, signaling, and metabolic pathways. Cell Metab. (2012) 16:153–66. 10.1016/j.cmet.2012.06.011 22795476

[6] Baracos VE Martin L Korc M Guttridge DC Fearon KCH. Cancer-associated cachexia. Nat Rev Dis Primers. (2018) 4:17105. 10.1038/nrdp.2017.105 29345251

[7] Seelaender M. Fish oil supplementation and cancer cachexia. In: Fish and Fish Oil in Health and Disease Prevention. Elsevier (2016). p. 283–9.

[8] Calder PC. Omega-3 fatty acids and inflammatory processes. Nutrients. (2010) 2:355–74. 10.3390/nu2030355 22254027 PMC3257651

[9] Mocellin MC Camargo CQ Nunes EA Fiates GMR Trindade EBSM. A systematic review and meta-analysis of the n-3 polyunsaturated fatty acids effects on inflammatory markers in colorectal cancer. Clin Nutr. (2016) 35:359–69. 10.1016/j.clnu.2015.04.013 25982417

[10] Laviano A Seelaender M Sanchez-Lara K Gioulbasanis I Molfino A Fanelli FR. Beyond anorexia -cachexia. Nutrition and modulation of cancer patients' metabolism: Supplementary, complementary or alternative anti-neoplastic therapy?. Eur J Pharmacol. (2011) 668 (Suppl 1):S87–90. 10.1016/j.ejphar.2011.06.060 21810420

[11] Arends J Bachmann P Baracos V Barthelemy N Bertz H Bozzetti F . ESPEN guidelines on nutrition in cancer patients. Clin Nutr. (2017) 36:11–48. 10.1016/j.clnu.2016.07.015 27637832

[12] Higgins JPT Thomas J Chandler J Cumpston M Li T Page MJ Welch VA (editors). Cochrane Handbook for Systematic Reviews of Interventions version 6.2 (updated February 2021). Cochrane (2021). Available online at: www.training.cochrane.org/handbook

[13] Moher D Liberati A Tetzlaff J Altman DG. Preferred reporting items for systematic reviews and meta-analyses: the PRISMA statement. PLoS Med. (2009) 6:e1000097. 10.1371/journal.pmed.1000097 19621072 PMC2707599

[14] Higgins JPT Altman DG Gotzsche PC Juni P Moher D Oxman AD . The Cochrane Collaboration's tool for assessing risk of bias in randomised trials. BMJ. (2011) 343:d5928. 10.1136/bmj.d5928 22008217 PMC3196245

[15] Review manager (RevMan). [Computer program]. Version 5.4. The Cochrane Collaboration (2020).

[16] Wan X Wang W Liu J Tong T. Estimating the sample mean and standard deviation from the sample size, median, range and/or interquartile range. BMC Med Res Methodol. (2014) 14:135. 10.1186/1471-2288-14-135 25524443 PMC4383202

[17] Carvalho TC Cruz BCS Viana MS Martucci RB Saraiva DCA Reis PF. Effect of nutritional supplementation enriched with eicosapentaenoic acid on inflammatory profile of patients with oral cavity cancer in antineoplastic pretreatment: a controlled and randomized clinical trial. Nutr Cancer. (2017) 69:428–35. 10.1080/01635581.2017.1274406 28128983

[18] Faber J Uitdehaag MJ Spaander M van Steenbergen-Langeveld S Vos P Berkhout M . Improved body weight and performance status and reduced serum PGE 2 levels after nutritional intervention with a specific medical food in newly diagnosed patients with esophageal cancer or adenocarcinoma of the gastro-esophageal junction. J Cachexia Sarcopenia Muscle. (2015) 6:32–44. 10.1002/jcsm.12009 26136410 PMC4435095

[19] Liu Y Jia Z Dong L Wang R Qiu G. A randomized pilot study of atractylenolide I on gastric cancer cachexia patients. Evid Based Complement Alternat Med. (2008) 5:337–44. 10.1093/ecam/nem031 18830451 PMC2529387

[20] Persson C Glimelius B Rönnelid J Nygren P. Impact of fish oil and melatonin on cachexia in patients with advanced gastrointestinal cancer: a randomized pilot study. Nutrition. (2005) 21:170–8. 10.1016/j.nut.2004.05.026 15723745

[21] Solís-Martínez O Plasa-Carvalho V Phillips-Sixtos G Trujillo-Cabrera Y Hernández-Cuellar A Queipo-García GE . Effect of eicosapentaenoic acid on body composition and inflammation markers in patients with head and neck squamous cell cancer from a public hospital in Mexico. Nutr Cancer. (2018) 70:663–70. 10.1080/01635581.2018.1460678 29697274

[22] Yeh KY Wang HM Chang JWC Huang JS Lai CH Lan YJ . Omega-3 fatty acid-, micronutrient-, and probiotic-enriched nutrition helps body weight stabilization in head and neck cancer cachexia. Oral Surg Oral Med Oral Pathol Oral Radiol. (2013) 116:41–8. 10.1016/j.oooo.2013.01.015 23562359

[23] Newell M Mazurak V Postovit LM Field CJ. N-3 long-chain polyunsaturated fatty acids, eicosapentaenoic and docosahexaenoic acid, and the role of supplementation during cancer treatment: a scoping review of current clinical evidence. Cancers (Basel). (2021) 13:1206. 10.3390/cancers13061206 33801979 PMC8000768

[24] Ding XZ Hennig R Adrian TE. Lipoxygenase and cyclooxygenase metabolism: new insights in treatment and chemoprevention of pancreatic cancer. Mol Cancer. (2003) 2:1–12. 10.1186/1476-4598-2-10 12575899 PMC149414

[25] PC C. Omega-3 fatty acids and inflammatory processes: from molecules to man. Biochem Soc Trans. (2017) 45:1105–15. 10.1042/BST20160474 28900017

[26] Calder PC. Omega-3 polyunsaturated fatty acids and inflammatory processes: nutrition or pharmacology? Br J Clin Pharmacol. (2013) 75:645–62. 10.1111/j.1365-2125.2012.04374.x 22765297 PMC3575932

[27] van Norren K Kegler D Argilés JM Luiking Y Gorselink M Laviano A . Dietary supplementation with a specific combination of high protein, leucine, and fish oil improves muscle function and daily activity in tumour-bearing cachectic mice. Br J Cancer. (2009) 100:713–22. 10.1038/sj.bjc.6604905 19259092 PMC2653763

[28] Deutz NEP Safar A Schutzler S Memelink R Ferrando A Spencer H . Muscle protein synthesis in cancer patients can be stimulated with a specially formulated medical food. Clin Nutr. (2011) 30:759–68. 10.1016/j.clnu.2011.05.008 21683485 PMC3964623

[29] Marventano S Kolacz P Castellano S Galvano F Buscemi S Mistretta A . A review of recent evidence in human studies of n-3 and n-6 PUFA intake on cardiovascular disease, cancer, and depressive disorders: does the ratio really matter? Int J Food Sci Nutr. (2015) 66:611–22. 10.3109/09637486.2015.1077790 26307560

[30] Nindrea RD Aryandono T Lazuardi L Dwiprahasto I. Association of dietary intake ratio of n-3/n-6 polyunsaturated fatty acids with breast cancer risk in Western and Asian Countries: a meta-analysis. Asian Pac J Cancer Prev. (2019) 20:1321. 10.31557/APJCP.2019.20.5.1321 31127884 PMC6857870

